# Why 6 Fingers Are Better Than 5! The Versatility and Utility of the Sixth Finger Knot Pusher in Arthroscopic Shoulder Surgery

**DOI:** 10.1016/j.eats.2025.103675

**Published:** 2025-06-17

**Authors:** Mustafa S. Rashid, Georgios Mamarelis, Michele Novak, Ian K.Y. Lo

**Affiliations:** East Suffolk and North Essex NHS Foundation Trust, Colchester, UK; University of Calgary, Calgary, Alberta, Canada

## Abstract

Arthroscopic knot tying in shoulder surgery offers the surgeon versatility and security when performing certain procedures. While knotless techniques have become popular, the young surgeon should develop and maintain their knot-tying skills. The surgeon’s knot has been shown to offer the best loop and knot security on testing. The sixth finger knot pusher is a double-lumen knot pusher that permits tying the arthroscopic surgeon’s knot, while offering a number of advantages over a traditional knot pusher. Its utility in shoulder surgery, as well as its advantages and disadvantages, are discussed in this Technical Note.

In the golden era of innovation in arthroscopy, working with a talented engineer, Don Grafton, Dr. Stephen Burkhart developed the “sixth finger,” a double-lumen knot pusher. The premise of this device was to allow secure arthroscopic knot-tying. The challenge was that when tying knots, particularly in the rotator cuff, traditional knot pushers would often lead to slipping of the first throw and poor loop security. In an arthroscopic surgeon’s knot, subsequent alternating half-hitches would lock the initial loop. The sixth finger permits the surgeon to maintain constant tension on the initial loop while sliding the alternating half-hitches to lock the knot. This has been demonstrated to show superior loop and knot security on testing.^1^ Surgeons also developed alternative methods of arthroscopic knot-tying, including sliding and sliding locking arthroscopic knots, but the surgeon’s knot, backed up with alternating half-hitches, remains the gold standard for loop and knot security.^1^^,^^2^

Many studies have tried to investigate the performance of knotted versus knotless repairs, focusing on biomechanical testing and, occasionally, on clinical performance.^3^^,^^4^ While the majority of biomechanical testing revealed better compression of the tissues, most clinical studies did not show a significant difference. Given that the costs are similar and knotless repairs are technically easier and quicker to perform, many surgeons moved toward a knotless repair.^5^ However, we believe that surgeons should remain able and competent to tie arthroscopic knots, as this skill is still required in a variety of situations where knotless repair is insufficient. The article outlines the rationale, benefits, and technical considerations of using the sixth finger knot pusher (Arthrex) to manipulate tissue, secure knot stacks, and conduct a wide range of clinical applications with this device in arthroscopic shoulder surgery.

## Surgical Technique

**Fig 1:**
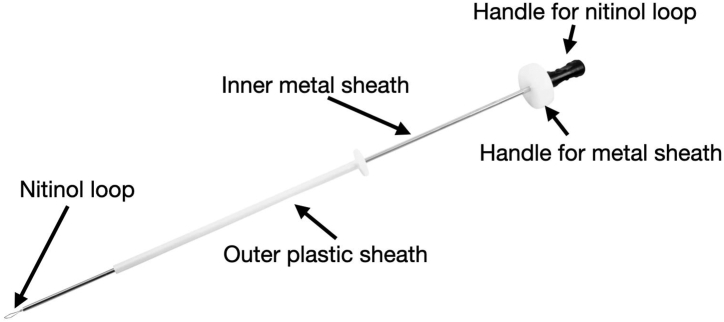
The sixth finger knot pusher (Arthrex).

The sixth finger knot pusher (Arthrex) has 3 components: a nitinol loop, an inner metal sheath, and a plastic outer sheath (Fig 1). The post limb of the suture is passed through the inner metal lumen by shuttling it, using the loop of the nitinol wire. The surgeon then secures the post limb with their dominant hand. A simple underhand throw is performed on the non–post limb, which is then passed into the joint using the outer plastic sheath. This initial loop is tensioned with tactile feedback by pulling up on the post limb while pushing down on the metal sheath. The plastic sheath is pulled back outside the shoulder. Next, an underhand throw is passed on the non–post limb. On this occasion, loop security is maintained by the surgeon’s right hand, which is pulling on the post limb and pushing down on the metal inner sheath of the knot pusher. The outer plastic sheath passes the half-hitch into the shoulder, laying it on top of the surgeon’s knot. The surgeon then past-points using the metal sheath of the knot pusher to tension this throw. After doing so, the metal sheath is placed back on the knot, and tension is maintained. The process is then repeated to create a surgeon’s knot with 3 underhand throws on top of one another. To lock the knot, 3 alternating half-hitches are placed. Alternating the direction of the throws (underhand and overhand) can be done, or the surgeon may choose to use only underhand throws accompanied by “flipping the post” on throws 4 and 6 (Video 1). Pearls and pitfalls are outlined in Table 1.

**Table 1:** Pearls and Pitfalls of Tying Arthroscopic Knots Using the Sixth Finger Knot Pusher (Arthrex)

Pearls	Pitfalls
After passing the first loop into the shoulder, regrasp the post limb at the top of the sixth finger to tension this loop appropriately.	Not maintaining tension on the first loop will result in a slack knot, irrespective of how tightly the subsequent throws are tensioned.
Use the end of the inner metal lumen to manipulate the tissues being tied and to ensure the knot stack is placed at the desired location.	Despite the radial slit on the circular disc, it is not recommended to use this to park the post limb suture. This is because the tensioning of the first loop is dynamic and not static. The surgeon should pay attention to the amount of tension imparted on the loop.
When passing the half-hitches into the shoulder, ensure the non–post limb is aligned to the metal sheath. If this limb is wrapped around the metal sheath, rotate both the circular disc and the plastic outer sheath in the direction to unwrap this limb. Doing this will improve the density of the knot stack.	If the end of the metal lumen is moved away from the tissue being tied, there is a chance the tissue will be manipulated into an undesired position.
For throws 4 and 6, you may either use underhand throws and flip the post or use an overhand throw and maintain the same post throughout.	By not aligning the non–post limb throws as they are passed onto the knot stack, the knot stack will not sit tightly and compact on the tissue.
When maintaining tension on the post limb, wrap the limb around the little and/or ring finger, freeing up your thumb and index finger to push on the circular disc to provide countertension.	When passing the non–post limb throws into the shoulder, if there is no tension on this hitch, then the plastic sheath will not be able to push the suture into the shoulder.

**Fig 2:**
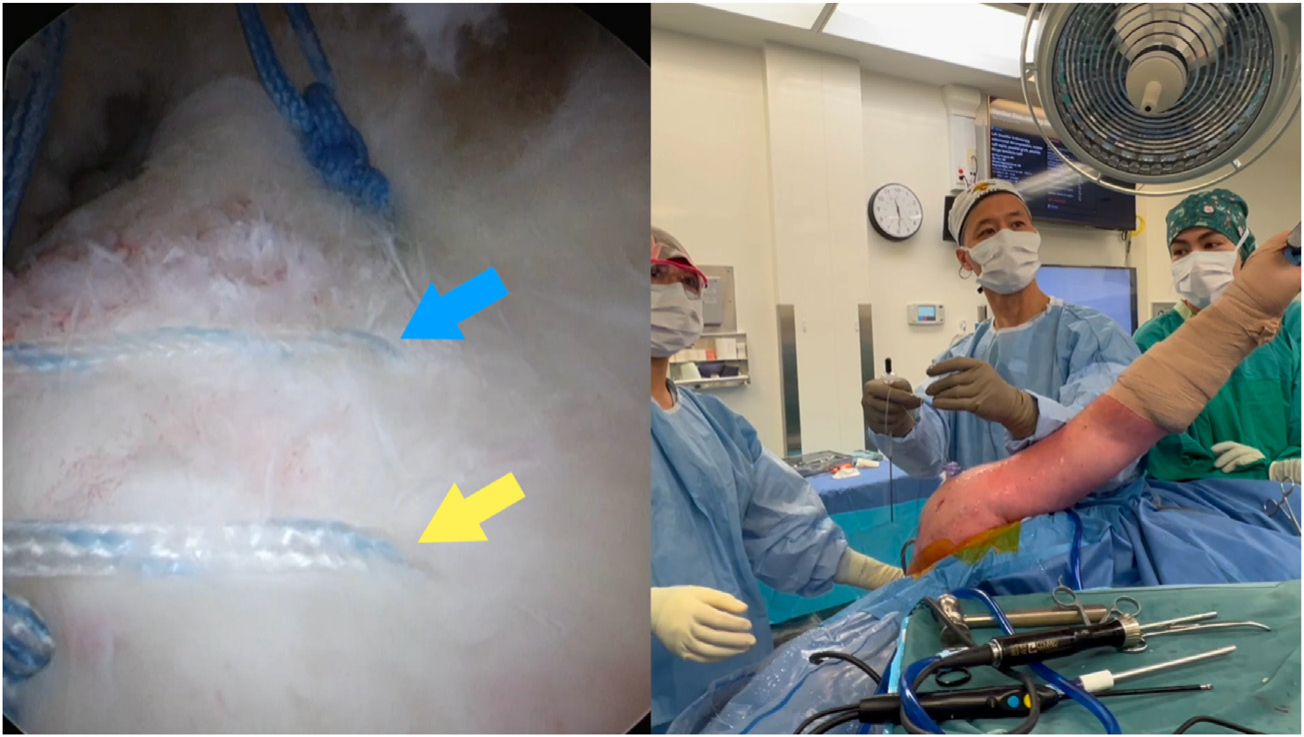
Identify which limb will be the post limb. In this example, the blue and yellow arrows denote 2 limbs (passed in a horizontal mattress configuration) from the same pair of sutures coming from a posteromedial anchor. Viewing portal: posterior on the left shoulder in a lateral decubitus position.

**Fig 3:**
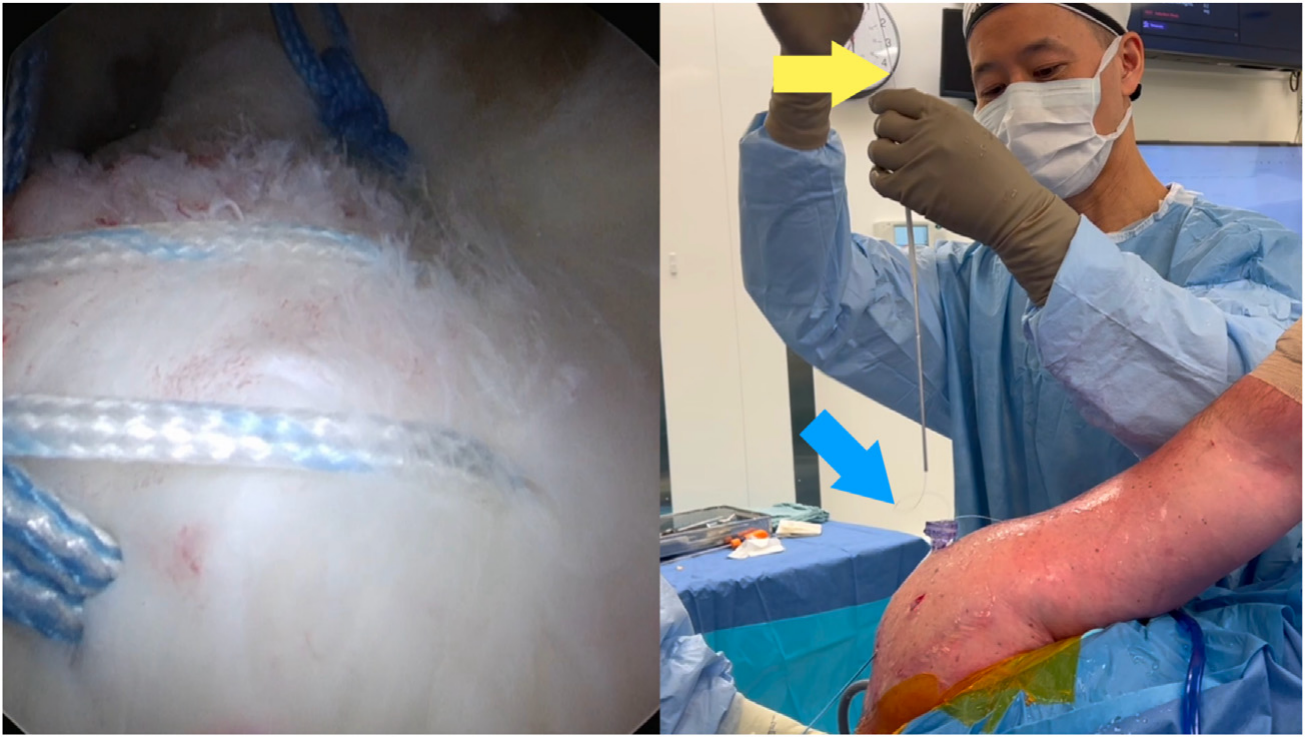
Viewing portal: posterior on the left shoulder in a lateral decubitus position. Use the nitinol loop (yellow arrow) through the metal lumen of the sixth finger knot pusher (Arthrex) to shuttle the designated post limb suture through the inner metal sheath.

**Fig 4:**
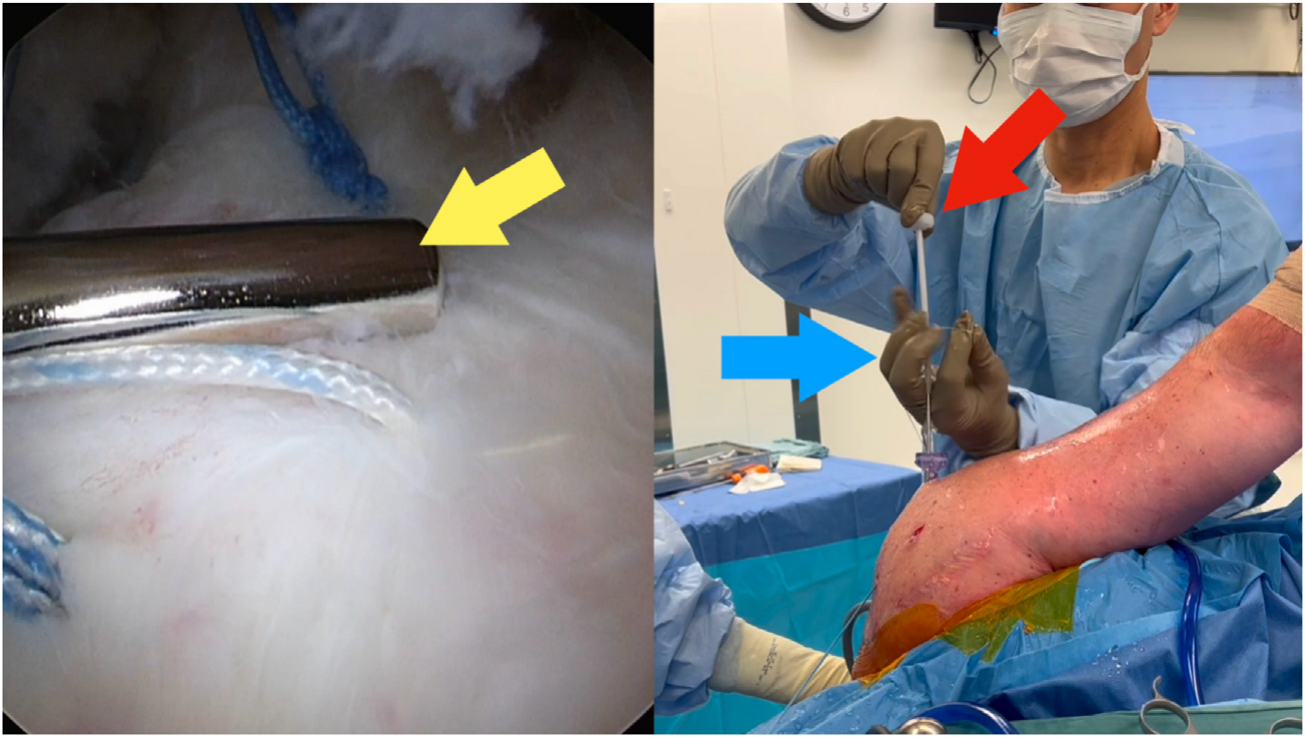
Viewing portal: posterior on the left shoulder in a lateral decubitus position. Keep the end of the inner metal lumen of the sixth finger knot pusher (Arthrex) on the tissue (yellow arrow) using your right hand by pushing down on the handle (red arrow). Using your left hand, pass a single underhand throw (blue arrow) around the metal sheath extracorporeally.

**Fig 5:**
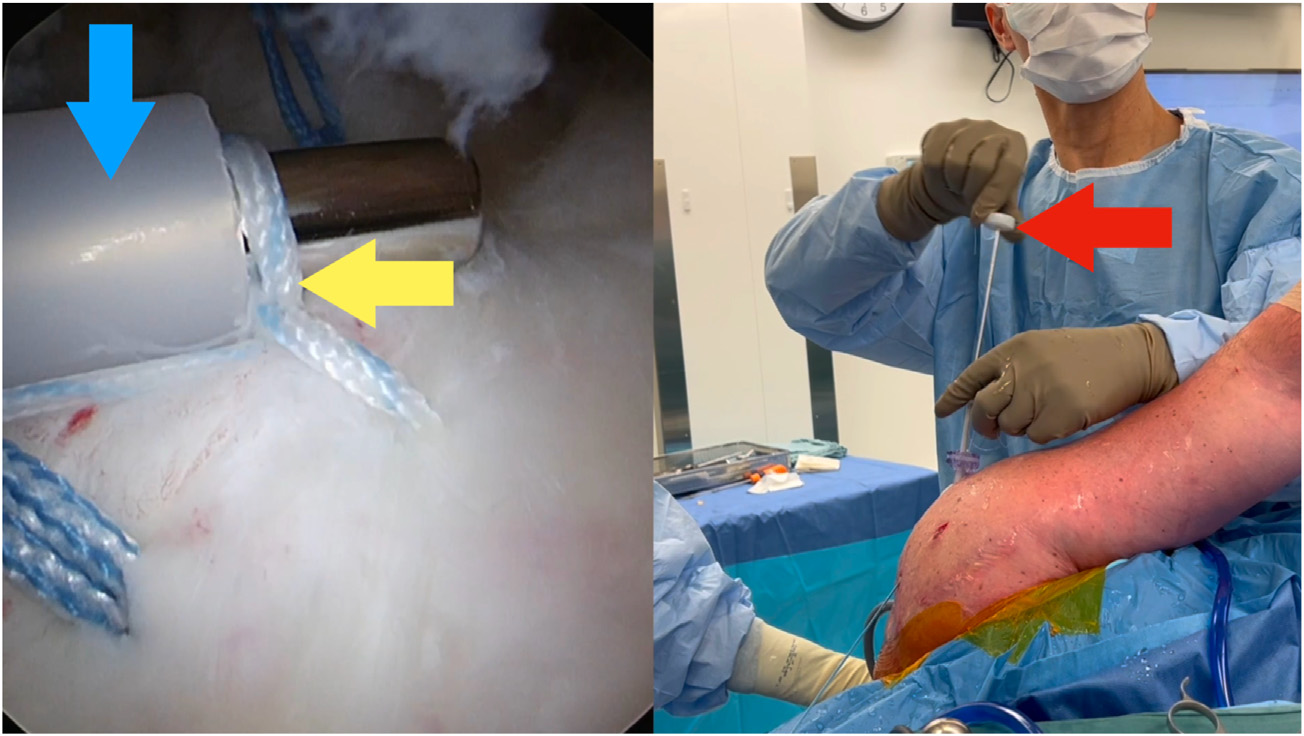
Viewing portal: posterior on the left shoulder in a lateral decubitus position. Push the throw (yellow arrow) into the shoulder by pushing the outer plastic sheath (blue arrow). Maintain tension on the suture by pushing down on the handle (red arrow).

**Fig 6:**
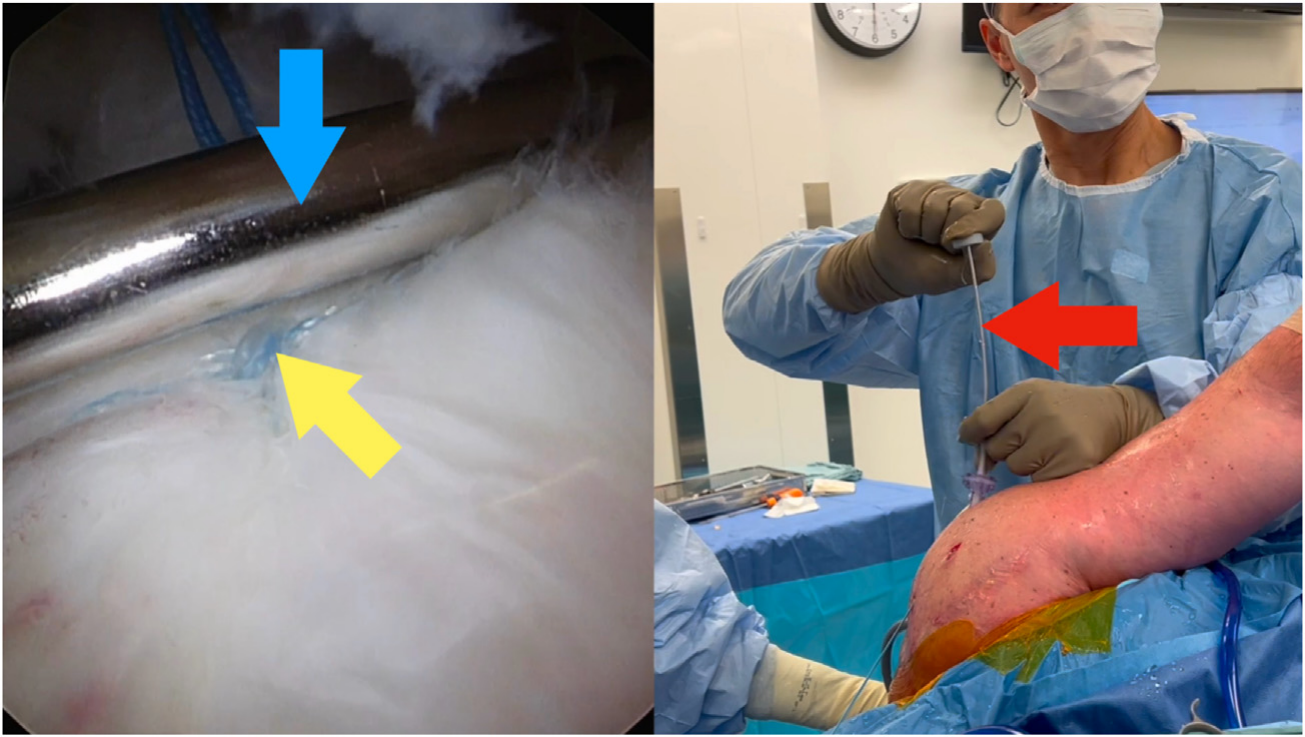
Viewing portal: posterior on the left shoulder in a lateral decubitus position. Use the inner metal sheath to past-point the suture (blue arrow) beyond the knot (yellow arrow). The metal sheath will bend slightly as shown (red arrow). Use your left hand to pull up on the other limb to provide countertraction.

### Step-by-Step Guide

1. Identify which limb is to be the post limb (Fig 2). Pass the end of this limb in the nitinol loop and pull through the inner lumen of the knot pusher (Fig 3).
2. Grasp this limb at the top end of the knot pusher and wrap around a finger. Hold the circular disc of the knot pusher with the same hand (left hand for right hand–dominant surgeons).
3. Take the non–post limb in your right hand and throw an underhand throw around the knot pusher (Fig 4). The plastic outer sheath should be at the top of the device, and so this throw should wrap around the inner metal sheath of the knot pusher, next to the arthroscopic cannula (recommended).
4. Keeping tension on the non–post limb, use the outer plastic sheath to push the throw into the shoulder (Fig 5). Push the throw off the end of the metal sheath. Retension this loop by pulling on the post limb and pushing on the circular disc of the knot pusher. This will also put tension on the tissue being tied down, creating compression.
5. Repeat steps 3 to 4 two more times to create 3 underhand throws on top of one another. Tension each throw by past-pointing beyond the knot (Fig 6).
6. To lock the knot, use the outer plastic sheath to push an overhand throw into the shoulder. Push the throw off the end of the metal sheath and past-point to tension the knot. An alternative to the overhand throw is to “flip the post.” This is done by changing the tension from the post limb to the non–post limb and past-pointing the post limb.
7. Repeat steps 3 to 4 one more time to back up the 3 underhand throws and 1 overhand throw with a fourth underhand throw.
8. Repeat step 6 to complete the surgeon’s knot with a final (sixth) throw. This final throw is an overhand throw, or with an underhand throw and a flipped post.
9. The final knot should have 6 throws with an alternating post or alternating half-hitch direction at throws 4 and 6.

This arthroscopic knot-tying technique offers several advantages (Table 2). Maintaining tension throughout ensures high loop security. Continuous tactile feedback helps prevent strangulation and overtensioning. Rotating both sheaths of the knot pusher aligns the half-hitch, minimizing suture wrapping and enhancing security.

**Table 2:** Advantages and Disadvantages of Utilizing the Sixth Finger Knot Pusher to Tie Arthroscopic Knots

Advantages	Disadvantages
Using the sixth finger knot pusher provides the best loop and knot security for arthroscopic knots.	Learning to use the sixth finger knot pusher can be challenging for surgeons with little or no experience in arthroscopic knot-tying.
The knot pusher acts like an extension of the surgeon’s finger, inside the shoulder, allowing them to manipulate the tissues being tied to a desirable position.	The device allows the surgeon to tension the knot stack significantly more than a traditional knot pusher, and care must be taken not to overtension the knot.
Becoming proficient in tying arthroscopic knots using this device allows the surgeon to match the repair construct to the pathology better, thereby moving away from a one-size-fits-all approach.	Some surgeons may find that certain high-tensile-strength ultra-high molecular weight polyethylene sutures will cut their fingers, despite wearing 2 layers of surgical gloves.
When tying locking sliding arthroscopic knots, such as Nicky’s knot, Duncan loop, Weston knot, or SMC knots, the surgeon pulls 1 suture limb to bring the knot down onto the tissue. In doing so, the tissue is being brought away from where it is intended to be. The surgeon is also placing undue tension on the suture anchor (if being used), which may fail in weak bone.	With practice, surgeons tying arthroscopic knots using the sixth finger knot pusher can become swift and proficient, but it will usually add more surgical time than a knotless approach.

SMC, Samsung Medical Centre.

The sixth finger knot pusher (Arthrex) also aids in tissue and knot stack manipulation. It allows precise positioning of tied tissue and repositioning of the knot stack, such as away from cartilage. By placing the metal sheath tip over the target area and tensioning the post limb, the surgeon directly influences knot placement.

## Discussion

The sixth finger knot pusher represents a device that permits the gold standard for arthroscopic knot-tying. While it may be desirable to adopt knotless techniques, surgeons should develop and maintain arthroscopic knot-tying skills to optimize the repair construct to the pathology, tissue characteristics, and surgical situation at hand. This device, albeit one of the earliest arthroscopic instruments developed for arthroscopic knot-tying, has many advantages, including permitting tactile feedback when tying friable tissues, manipulating tissues to optimal positions before securing the repair, and placing knot stacks away from cartilage surfaces.

From a biomechanical perspective, knotless repairs may perform similarly to knotted repairs in some circumstances. There may even be some specific scenarios when a knotless repair is preferred. One such example is in primary labral repair surgery for traumatic anterior shoulder instability. Assuming the capsulolabral tissues are normal, a knotless repair of good-quality tissue allows for repair without any knot stacks, which may irritate the humeral head cartilage. However, this type of knotless repair, irrespective of which type of knotless anchor is used, relies on visual feedback to determine appropriate tension of the repair. In a similar situation, but where the quality of the capsulolabral tissue is poor, visual feedback regarding tensioning may result in “cheese-wiring” of the suture through the tissue. In this scenario, tying knots allows the surgeon to feel the optimal tension, thereby reducing the risk of cut-through of the suture.

Similarly, in a small full-thickness rotator cuff repair, many surgeons opt to pass a horizontal mattress suture configuration into a single knotless anchor. Here, little compression of the footprint is achieved, although the advantage is that it is quick and simple to perform the repair. To optimize healing, a secure hold within the tendon edge is required, alongside adequate compression of the footprint and the absence of gapping at the tendon-bone interface. All 3 parameters can be better achieved with a double-row repair and medial row knot-tying, facilitated by the sixth finger knot pusher. In larger tears, it is important for the surgeon to appreciate the degree of tension on the repair. Many surgeons may wish to medialize the footprint and perform a single-row repair, rather than forcing the retracted and large tear into a knotless double-row repair. In this situation, tying secure knots is helpful to maximize the chances of healing.

In summary, the sixth finger knot pusher (Arthrex) allows optimal loop and knot security to tie an arthroscopic surgeon’s knot backed up with alternating half-hitches. Its utility allows the surgeon tactile feedback regarding soft tissues being repaired and subtle manipulation of tissues, grafts, and knot stacks.

## Disclosures

The authors declare the following financial interests/personal relationships which may be considered as potential competing interests: M.S.R. has received speaking and lecture fees from 10.13039/100009026Smith & Nephew and Limacorporate Spa. I.K.Y.L. reports a relationship with Smith & Nephew that includes consulting or advisory, funding grants, speaking and lecture fees, and travel reimbursement. All other authors (G.M., M.N.) declare that they have no known competing financial interests or personal relationships that could have appeared to influence the work reported in this paper.

## Supplementary Data

Video 1 Video showing how to utilize the sixth finger knot pusher in arthroscopic shoulder surgery, including technical pearls for “flipping the post” and improving knot security.
